# Age-Related Changes in the Hepatic Pharmacology and Toxicology of Paracetamol

**DOI:** 10.1155/2011/624156

**Published:** 2011-05-11

**Authors:** Sarah J. Mitchell, Alice E. Kane, Sarah N. Hilmer

**Affiliations:** ^1^Laboratory of Experimental Gerontology, National Institute on Aging, National Institutes of Health, Baltimore, MD 21224-6825, USA; ^2^Kolling Institute of Medical Research, The Royal North Shore Hospital, St. Leonards, NSW 2065, Sydney, Australia; ^3^Department of Clinical Pharmacology, The Royal North Shore Hospital, Pacific highway, St. Leonards, NSW 2065, Sydney, Australia; ^4^Sydney Medical School, University of Sydney, St. Leonards, NSW 2065, Sydney, Australia; ^5^Laboratory of Ageing and Pharmacology, Level 12 Kolling Building, The Royal North Shore Hospital, Pacific Highway, St Leonards NSW 2065, Australia

## Abstract

Optimal pharmacotherapy is determined when the pharmacokinetics and pharmacodynamics of the drug are understood. However, the age-related changes in pharmacokinetics and pharmacodynamics, as well as the increased interindividual variation mean optimal dose selection are a challenge for prescribing in older adults. Poor understanding of how hepatic clearance and toxicity are different with age results in suboptimal dose selection, poor efficacy, and/or increased toxicity. Of particular concern is the analgesic paracetamol which has been in use for more than 50 years and is consumed by a large proportion of older adults. Paracetamol is considered to be a relatively safe drug; however, caution must be taken because of its potential for toxicity. Paracetamol-induced liver injury from accidental overdose accounts for up to 55% of cases in older adults. Better understanding of how age affects the hepatic clearance and toxicity of drugs will contribute to evidence-based prescribing for older people, leading to fewer adverse drug reactions without loss of benefit.

## 1. Introduction

Paracetamol remains one of the most studied agents that cause hepatotoxicity due to its clinical relevance and to its dose-dependent hepatotoxicity in animals and humans [1]. Paracetamol is an effective analgesic agent and represents the first-line analgesic therapy for nonmalignant pain [2]. However, the use of paracetamol is limited by its potential to cause hepatotoxicity. With old age, there is an increase in disease for which medications may provide benefit; however, the incidence of serious adverse drug reactions (ADRs) also increases with increasing age, even after controlling for increased medication use [3]. In older adults, most ADRs, including drug-induced liver injury (DILI), are dose-related [4]. Therefore, optimising the safety and efficacy of medication use in older adults is important. 

**Figure 1:**
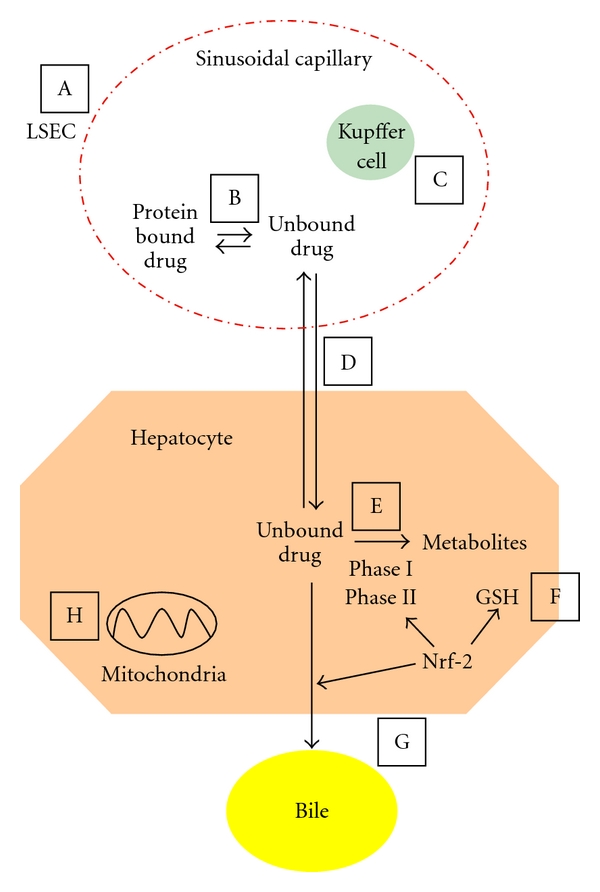
Hepatic pharmacology and toxicology in old age. (A) Pseudocapillarisation (thickening, defenestration, and basement membrane formation) of the liver sinusoidal endothelial cells (LSECs) may affect susceptibility to drug-induced liver injury (DILI); (B) Changes in protein binding in old age affect the amount of free drug available for clearance; (C) Dysregulation of Kupffer cell activation may alter inflammatory response to DILI; (D) Pseudocapillarisation of the LSECs, and any changes in transporters, may alter drug transfer from the blood to hepatocytes; (E) Age-related changes in hepatic metabolism affect drug clearance: phase I metabolism is reduced, and changes in phase II metabolism are less well understood; (F) Reduced glutathione (GSH) in old age increases injury by toxic metabolites; (G) Expression of hepatic transporters in response to drug toxicity is poorly described in old age and affects biliary excretion of drugs and their metabolites; (H) Changes in mitochondrial structure and function in old age alter response to reactive oxygen species and cell death pathways. Steps (E), (F), and (G) are regulated by nuclear factor E2-related factor 2 (Nrf-2) which has reduced hepatic expression in old age. Figure adapted from [24, 25].

For most drugs, the evidence base for dose adjustment in older people is limited to pharmacokinetic studies in small populations of healthy volunteers. There is very little data available on the clinical outcomes of dose adjustment, particularly in the frail aged. In all age groups, an important susceptibility factor for hepatotoxicity is genetic variability [5]. In older people, this may be compounded by the multi-factorial large interindividual variation in response to medications further increasing the risks of toxicity and poor efficacy [5]. This is a particular concern in frailty, a condition of increased vulnerability to adverse events [6]. Although monitoring for clinical response is essential to optimise efficacy and reduce toxicity, the detection of adverse effects of medications in older patients may be complicated by nonspecific presentation as “geriatric syndromes” [7]. In addition, age-related changes in the pharmacokinetics and pharmacodynamics of drugs further compounds the risk of toxicity. In older adults, the clinical increased risk of paracetamol hepatotoxicity is likely to be related to dosing that does not account for decreased liver volume with age, and to frailty and malnutrition [8]. However, recent discoveries about the ageing liver identify novel mechanisms for age-related changes in hepatic pharmacology and toxicology (summarised in Figure 1). In this paper, we describe the age-related physiologicalchanges, with particular attention to the liver specific changes, and how they can impact on hepatic pharmacology and toxicology of paracetamol.

## 2. Physiological Changes in Ageing

The most marked pharmacologic change with ageing is increased interindividual variation. The most significant pharmacokinetic change in ageing is related to the decreased hepatic mass, uptake, and blood flow [9, 10] and to decreased renal function [11], impairing the clearance of many drugs and their metabolites [12].  Table 1 summarises the physiological changes associated with ageing and frailty that can impact on the pharmacokinetics and pharmacodynamics of drugs. 

Changes in the pharmacodynamics of drugs in old age is related to changes in drug receptors, physiologic reserve and in response to injury [12]. However, these changes have not been as well characterised in ageing as the pharmacokinetic changes. The cardiovascular and central nervous systems are the two best described. A reduction in the responsiveness of the cardiac and *β*-adrenergic system has been observed in older adults [14]. In the central nervous system, the numbers of dopaminergic neurons and dopamine D2 receptors decrease with age resulting in extrapyramidal side effects [26]. Studies in animal models suggest that increased sensitivity to narcotic and anaesthetic agents may be due to alteration of opioid receptors (decreased *μ*-opioid receptor density and increased affinity) in old age [15]. 

Ageing is associated with changes in body composition, including a reduction in total and lean body mass, and a relative increase in fat mass [12, 27], which may affect the volume of distribution and loading dose of drugs. Sarcopenia, defined as loss of muscle mass and strength with ageing, increases with age and is associated with frailty [5, 27]. It can be the result of concomitant diseases, neuroendocrine dysregulation and/or chronic inflammation [27].

## 3. Liver Specific Changes in Ageing

The age-related reduction in liver size is noted to be in the order of 25 to 35%, which has been confirmed in many species including humans [9, 28–30]. The main age-related change in the physiology of the liver is a substantial reduction in blood flow of about 40% [29] which has been postulated to be due to leukocyte accumulation in the sinusoids and narrowing of sinusoidal lumens due to pseudocapillarisation and dysfunction of the liver sinusoidal endothelial cells (LSECs) [31]. 

### 3.1. Pseudocapillarisation of the Liver Sinusoidal Endothelium

The sinusoidal endothelium is a thin fenestrated endothelium lacking a basal lamina and punctuated with fenestrations of 50 to 200 nm in diameter grouped together in clusters known as liver sieve plates [32–35]. With ageing, the LSECs undergo ultrastructural changes termed pseudocapillarisation [16] that include that include loss of fenestrations, thickening of the endothelium, perisinusoidal collagen deposition, and basal lamina formation [16, 36, 37], which may affect hepatic drug disposition. Recently, the endocytic capacity of LSECs was reported to be reduced in old age, which may be especially important in situations with increased circulatory waste loads [38].

### 3.2. Dysregulation of Kupffer Cell Activation

Kupffer cells (KCs) are the resident phagocytic macrophages in the liver, which represent the largest population of fixed macrophages in the body and account for approximately 20% of nonparenchymal cells in the liver [39]. KCs have diverse functions, including phagocytosis, endocytosis, immunomodulation, and synthesis and secretion of numerous biologically active mediators [39, 40]. Furthermore, the dual role of activated KCs in releasing both pro- and anti-flammatory mediators during the different stages of liver injury and regeneration has been demonstrated [39]. It is likely that dysregulation of the KCs with ageing may alter the inflammatory response to DILI; however, there is conflicting evidence. For example, while there is a basal increase in the numbers of KCs in old rats [40], their activation in response to toxic doses of cadmium and endotoxin is decreased [41, 42]. However, KCs in lipopolysaccharide-treated had showed no difference in activity with the phagocytosis of fluorescent beads being similar across age groups [43]. The role of KCs in paracetamol-induced hepatotoxicity will be discussed below.

### 3.3. Age-Related Changes in Hepatic Metabolism Affect Drug Clearance

Age-related changes in hepatic metabolism will affect drug clearance and toxicity. In general, there is a reduction in Phase I metabolism in vivo with normal ageing in the order of 30%–50% [24]. Phase II metabolism appears to be maintained in the healthy elderly but reduced in the frail [24]. The Phase III transporters have not been well described in humans. 

The Phase I drug metabolising enzymes (DMEs) consist of the superfamily of CYP450 enzymes. Animal studies have indicated that total CYP content is reduced in aged rats [44]. In humans, it has been suggested that CYP450 content declines at a rate of 0.07 nmol/g of liver after 40 years of age [45]. However, there is conflicting evidence on the CYP activity with age (Table 2). For example evidence from liver biopsies of surgical patients indicate that normal ageing does not affect the activity of human CYP2E1, other evidence suggests that CYP2E1 activity may decrease with age [46]. Furthermore it has been suggested that the induction of CYP2E1 could also be affected by advanced age [47]. Interestingly it has shown that sex and concurrent medications have a greater effect than chronological age on CYP3A substrates in older patient populations [48, 49]. Frail older persons do not have slower erythromycin breath test results compared with non frail older persons potentially indicating preserved CYP3A activity as well as P-glycoprotein (P-gp) transport, in frailty [48].

 However, it can be assumed that there is some minor impairment in CYP activity with ageing given that there is a small decline (in the order of 20% observed in patients aged 25–75 years) in antipyrine clearance with age and antipyrine is metabolised by multiple CYPs [45, 50, 51]. The effect of disease, concurrent medications and frailty, and comorbidity on CYP activity still needs to be investigated given that these studies were conducted in relatively healthy volunteers. 

Phase II metabolism acts to increase hydrophilicity of the compounds, and thereby enhance excretion in bile and or urine [63]. Enzymes include the sulfotransferases, UDP-glucuronosyltransferases (UGTs), and glutathione s-transferases (GSTs). Most data suggest that the Phase II conjugation pathways are not altered by ageing [13, 52]. However, a recent reanalysis of pharmacokinetic studies in old age found that the apparent preservation of Phase II metabolism may have been confounded by inadequate consideration of protein binding [64]. Therefore, suggesting that Phase II metabolism is impaired in healthy ageing [64]. This is consistent with animal studies in which transcript profiles for the glucuronidation, sulfation, and glutathione conjugation genes are reportedly decreased in aged Fischer 344 rats [65].

The Phase III hepatic pathway encompasses the transporters on the basolateral and apical sides of the hepatocytes, which function to remove xenobiotics from the portal blood and to excrete them or their metabolites in to bile or blood, and includes the bile transporter P-gp [66–68]. The expression of hepatic transporters in response to drug toxicity is poorly described in old age and will affect biliary excretion of drugs and their metabolites. There are few human studies on Phase III hepatic metabolism in ageing. P-gp expression is increased in aged Fischer-344 male, but this is specific to the liver [69]. A small study of healthy volunteers found decreased P-gp activity in the blood brain barrier of five older healthy volunteers, age range 59–68 years [70]. It must be noted that the wide genetic interindividual variation in expression of P-gp [71] may be further be confounded by the increasing heterogeneity with age [67]. 

Ageing has been associated with decreased mRNA expression of organic anion-tran sporting polypeptide (Oatps) including Oatp1a1, Oatp1b2, and Oatp2b1, as well as organic cation transporters (Octs) Oct1, and sodium/taurocholate-cotransporting polypeptide in aged mice [53]. Furthermore, the mRNA expression of several efflux transporters including Multidrug resistant protein (Mrp)-2, Mrp6 and Mrp3 have been shown to be significantly reduced in old age in mouse livers [53]. However, what this means in terms of activity and how it translates to humans still needs to be determined.

### 3.4. Mitochondrial Structure and Function in Old Age

Changes in mitochondrial structure and function in old age [72] alter the response to reactive oxygen species and cell death pathways. It appears that malfunction and decrease of biogenesis of mitochondria seem to exert some of the most potent effects on the organism [72]; however, the exact mechanism still needs to be elucidated.

### 3.5. Glutathione in Old Age

Glutathione (GSH) has several important functions including detoxification of electrophiles, maintenance of essential thiol status of proteins and other molecules, scavenging of reactive oxygen species (ROS), providing cysteine as well as modulation of critical cellular processes such as DNA synthesis, microtubular-related processes, and immune function [73, 74]. These reactions are catalysed by the GSTs [74]. Hepatic GSH is decreased in aged rats [75, 76] and in aged mice [77, 78]. Serum GSH and the levels of its associated enzymes are decreased in ageing in humans [79]. In rats, this age-related decrease has been shown to be further exacerbated by ethanol consumption [80] which may be a problem in chronic alcoholics.

### 3.6. Nrf-2 in Old Age

Nuclear factor E2-related factor 2 (Nrf-2) regulates the transcription of antioxidant genes including genes for the Phase II conjugation enzymes (e.g., UGTs), glutathione homeostasis, stress response, and transporter proteins through the antioxidant-responsive element. Nrf-2 downregulation with ageing has been suggested as one mechanistic explanation for reduced Phase II metabolism in old age [81].

## 4. Implications of the Age-Related Alterations in Hepatic Pharmacology and Toxicology

**Figure 2:**
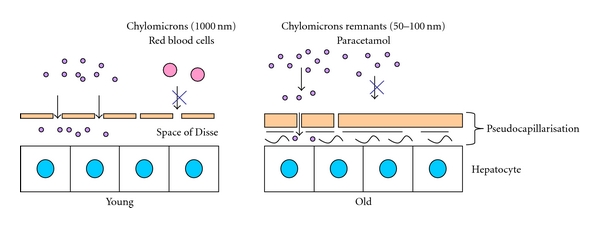
Age-related pseudocapillarisation of the liver sinusoid impairs the transfer of lipids (chylomicrons remnants) and paracetamol across the fenestrated liver sinusoidal endothelial cells (LSECs). Adapted from Le Couteur et al., 2002 (20).

A decline in liver volume and liver blood flow with ageing may be a major component of age-related alterations in the liver, leading to the fall in clearance of many of the drugs whose pharmacokinetics have been found to be altered with age [9]. Hepatic clearance is influenced by substrate delivery to the liver parenchymal cells and by the inherent metabolic capacity of the hepatocytes. Therefore, any change in the LSECs, including pseudocapillarisation, may alter drug transfer from the blood to hepatocytes. Age-related pseudocapillarisation has been shown to be associated with impaired transfer of lipoproteins as well as with paracetamol across the fenestrations [82, 83] as illustrated in Figure 2.

Changes in the inherent ability of the liver to detoxify toxic metabolites will lead to increased susceptibility to DILI. This may be due to the age-related dysfunction and reduced biogenesis of mitochondria [72], and/or the age-related reduction in Phase II metabolism and reduced hepatic GSH in old age [84], secondary to reduced transcriptional activity of Nrf-2 [81]. 

Interestingly “the mitochondrial hypothesis” implies that the gradual accumulation of initially silent mitochondrial injury which, when a critical threshold is reached, abruptly triggers liver injury [85]. This could explain why DILI does not affect all individuals equally (duration of exposure is not the same for all individuals), the delay in developing DILI by weeks or months (accumulation of deficits to reach a threshold), and why increasing age is a risk factor (due to duration of exposure or mitochondrial changes in ageing) [86]. The role of these changes and their effect on paracetamol-induced liver injury will be discussed below.

## 5. Paracetamol-Induced Liver Injuryin Ageing

Paracetamol (or acetaminophen) is a p-aminophenol derivative which was discovered at the John Hopkins University in 1877 [87]. Due to its safety profile, paracetamol is particularly useful in older adults; however, caution must be taken because of its potential for toxicity [88]. Paracetamol has the potential to cause liver damage and even liver failure in overdose and now case reports are emerging of people developing significantly increased ALT concentrations following therapeutic dosing [89], even in the absence of risk factors [89, 90]. Paracetamol causes dose-dependent hepatotoxicity through the metabolic bioactivation of the parent drug to toxic metabolite [1]. 

### 5.1. Epidemiology

A recent systematic investigation by the WHO Collaborating Centre for International Drug Monitoring reported that since 1969, paracetamol has been one of the five most common drugs associated with fatalities [91]. After 1990, there was a shift from halothane (immuno-allergic DILI) being the most common drug associated with a fatal outcome to paracetamol (dose-dependent DILI) [91, 92].  Table 3 shows selected reports of paracetamol-induced hepatotoxicity deaths and transplants, with special reference to those aged >60 years, in the United Kingdom, United States, Canada, Malaysia, and Australia for the period 1989–2010. In the United States, paracetamol is responsible for approximately half of the cases of acute liver failure [1].

### 5.2. Hepatotoxicity

At therapeutic doses paracetamol metabolised primarily in the liver to nontoxic metabolites via Phase II metabolism (conjugation) with glucuronide and sulphate, or cysteine [93]. A small amount of drug undergoes Phase I CYP450-mediated N-hydroxylation to form N-acetyl-p-amino-benzoquinone immine (NAPQI), a toxic metabolite [93–95]. The most important isoform responsible for this CYP450-mediated metabolism is CYP2E1, but CYP3A4 and CYP1A2 are also involved [93]. Under normal circumstances, NAPQI combines with sulphydryl groups in hepatic glutathione and is neutralized [93, 96]. The major conjugates, glucuronide and sulfate being more water-soluble than the parent drug, are both eliminated from the liver and blood mainly via the urine, with a small amount of the glucuronide conjugate eliminated via the bile [93, 97, 98].

Following ingestion of large amounts of paracetamol, conjugation pathways become saturated resulting in increased use of the CYP450 pathway, increased NAPQI formation and increased depletion of hepatic glutathione [97, 99]. The direct mechanism of paracetamol-induced liver injury involves the formation of the toxic metabolite, NAPQI, from paracetamol by the enzymes of the liver [100]. NAPQI can directly interact with macromolecules in the cell causing protein dysfunction, lipid peroxidation, damage of DNA, and oxidative stress [100]. Dysfunction of mitochondria may also result thereby in interrupting energy production and disrupting ionic gradients and intracellular calcium stores to result in cell death and liver damage [100, 101]. 

The formation of reactive metabolites such as NAPQI is an important initiating factor for DILI. It is the inflammatory immune response and the balance between the protective and toxic signalling processes of the cells involved in this response that determines the severity and progression of liver injury [102]. Holt and Ju (2006) suggest that hepatocyte stress or death, as a result of the reactive metabolite induced damage, causes the release of signals that stimulate activation of the innate immune cells of the liver [100]. KCs, natural killer cells and neutrophils, are part of this response [103] and are recruited and activated. These cells produce proinflammatory cytokines and mediators such as tumor necrosis factor (TNF)-*α*, interleukin (IL)-1*β* and interferon (IFN)-*γ* [104–106]. Other mediators released by these immune cells are protective and anti-inflammatory such as IL-10 [106] and IL-6 [107]. However, there is much disagreement between the studies, and the exact role of each of the cell types and mediators in DILI generally, as well as in paracetamol-induced liver injury, has yet to be fully determined [100].

### 5.3. The Role of Kupffer Cells in Paracetamol Induced Hepatotoxicity

Paracetamol-induced hepatotoxicity has been attributed in part to activation of KCs secondary to hepatocyte damage initiated by NAPQI [108, 109]. It is believed that Kupffer cell activation results in the release of a wide range of proinflammatory mediators capable of causing further hepatic injury [110]. However, there is controversial evidence surrounding the role of KCs in paracetamol-induced hepatotoxicity. The numbers of these F4/80 positive cells in the liver are increased following paracetamol treatment [111, 112]. Yet, macrophage depletion has been shown to have a role in both the protection [113] and potentiation of liver injury [114]. Pretreatment of rats with macrophage inactivators, such as gadolinium chloride and dextran sulfate, has been shown to decrease hepatic injury from paracetamol in rats [110, 115]. This was also observed in a mouse model of hepatotoxicity [113, 114] with the protection being ascribed to decreased formation of reactive oxygen and nitrogen species [113]. The use of liposome-encapsulated clodronate to deplete KCs from the liver [116] revealed a hepatoprotective role in a mouse model of paracetamol-induced liver injury [114]. Furthermore, the significant decrease in the levels of several cytokines and mediators, including IL-6-, IL-10-, and IL-18-binding protein may suggest that KCs mediate their beneficial role via the release of such soluble factors [114]. In support of this, it was recently suggested that a disturbance in the T-helper (Th)-1/Th-2 cytokine balance could play an important role in the pathogenesis of paracetamol-induced liver injury [117].

### 5.4. The Role of LSECS and Microvasculature Disturbance in Paracetamol Hepatotoxicity

It was recently suggested that the hepatoprotective role of KCs may be mediated, in part, via regulation of LSEC homeostasis and integrity [118]. In mice, the early events occurring in the hepatic microvasculature following paracetamol treatment include LSEC injury, which was exhibited by the swelling of LSECs, and the penetration of erythrocytes into the extra sinusoidal space [119]. Interestingly, these findings precede hepatocyte injury and suggest that the LSECs are a direct and early target during paracetamol hepatotoxicity [119, 120]. Furthermore, the structural and functional changes in LSECs could contribute to the initiation or progression of paracetamol-induced liver injury [119]. Taken together, this indicates that reduced sinusoidal perfusion and increased Kupffer cell activity participate in the development of liver injury elicited by paracetamol [119].

### 5.5. The Effect of Ageing on Susceptibility to Paracetamol-Induced Liver Injury

**Figure 3:**
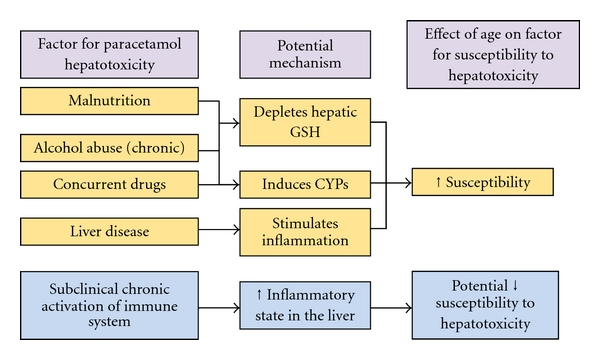
The effect of age on risk factors for paracetamol-induced hepatotoxicity and the potential mechanism through which they may act.

Risk factors for paracetamol hepatotoxicity include malnutrition which results in depletion of glutathione [121], chronic alcohol consumption, which acts to both reduce glutathione stores and induce CYP2E1 [122], and concurrent use of CYP-inducing drugs such as Isoniazid [123]. Inflammation as a result of bacterial or viral infection has also been identified as a risk factor for paracetamol hepatotoxicity [124] along with liver disease [125]. Interestingly, polymorphisms in the CYP2E1 enzyme causing altered acetylation status have been shown to be a factor influencing isoniazid hepatotoxicity [126, 127]. Conceivably, this may also be applicable to paracetamol with those individuals with a “rapid acetylator” phenotype may have accelerated production of the hepatotoxic NAPQI, however this needs to be substantiated further. Furthermore, the effect of age on these risk factors is not fully understood.  Figure 3 describes the risk factors for paracetamol hepatotoxicity and the effect of age as a modifier of the risk factor. Increasing age is associated with increased time to presentation [128], resulting in poorer outcome. Interestingly, in rats the risk of hepatotoxicity from paracetamol decreases with increasing age [129]. It also must be acknowledged that elevated liver enzymes after exposure to paracetamol have occurred in adults who have none of the reported risk factors for paracetamol toxicity [89, 130].

**Figure 4:**
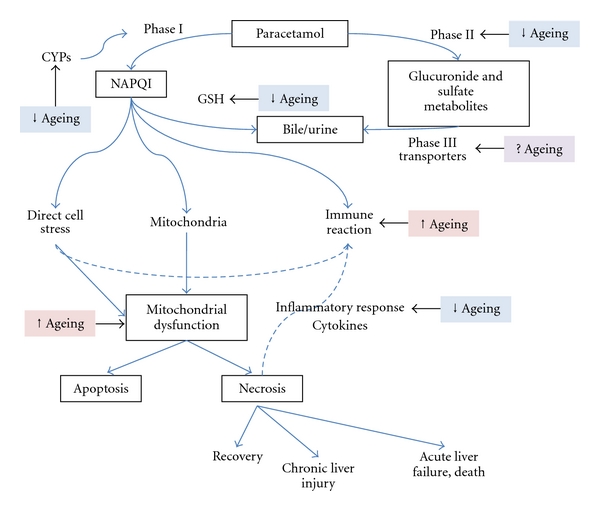
The effect of age on the hepatotoxic pathway for paracetamol-induced liver injury. At therapeutic doses, paracetamol metabolised primarily in the livervia the Phase II metabolism (conjugation). A small amount of drug undergoes Phase I CYP450- (CYP-)mediated N-hydroxylation to form N-acetyl-p-amino-benzoquinone immine (NAPQI), a toxic metabolite which is conjugated with hepatic glutathione (GSH) and is neutralised. The major metabolites are excreted via the urine or bile by Phase III transporters. Saturation of conjugation pathways results in increased use of the CYP450 pathway, increased NAPQI formation, and increased depletion of hepatic glutathione. NAPQI can cause injury through direct cell stress, direct mitochondrial inhibition, or through immune reactions. Initial injury leads to mitochondrial dysfunction leading to either apoptosis of damaged cells, or necrosis with recovery, chronic liver injury or actual liver failure, and death as potential outcomes. Additionally, necrosis can stimulate the inflammatory response leading to cytokine release and further potentiation of the immune reaction. Ageing can act at multiple parts of the pathway to either increase (↑) or decrease (↓) susceptibility to hepatotoxicity. It must be noted, however, that this is likely to vary between individuals. The effect of ageing on Phase III transporters is somewhat unknown (?) in humans. Picture adapted from Russmann et.al., 2009 [141].

The risk of hepatotoxicity from therapeutic doses of paracetamol in older people is not well defined. Older frail hospital in patients taking therapeutic paracetamol for five days do not have an increased risk of raised ALT compared to younger patient, although the clinical implications of such findings are not clear [131]. In people aged **≥** 65 years, the clinical increased risk of paracetamol hepatotoxicity is likely related to dosing that does not account for decreased liver volume with age, and to frailty and malnutrition [8]. Ageing and frailty are associated with a loss of reserves and increased state of vulnerability [132]. Therefore it is likely that the older frail patient will be at increased risk of DILI from therapeutic doses of medications. Changes in drug clearance in old age affect the formation and clearance of the toxic metabolites and therefore the susceptibility to DILI [67]. Interestingly one determinant of the variability in susceptibility to hepatotoxicity appears to be inflammatory stress [1]. Subclinical chronic activation of the immune system in older people [133] is likely to result in decreased response to injury.  Figure 4 summarises the paracetamol hepatotoxicity pathway and identifies potential parts of the pathway at which ageing may act to increase or decrease the susceptibility to toxicity. However, this is likely to vary between individuals.

### 5.6. Detection and Management of Paracetamol-Induced Liver Injury

Clinically, paracetamol overdose is associated with three main stages. The first lasts for approximately 24 hours and involves nonspecific gastrointestinal symptoms such as nausea, vomiting and abdominal pain with minimal elevation in serum liver enzyme concentrations. The second stage, from 24–72 hours, involves most notably the elevation of serum aspartate aminotransferase (AST) and alanine aminotransferase (ALT) concentrations released from damaged hepatocytes [134–136]. Serum ALT and total bilirubin are the most common biomarkers used to detect and manage hepatocellular injury [137]. Serum ALT is more liver specific than AST and is a very sensitive detector of hepatocellular necrosis; however, it cannot distinguish DILI from necrosis resulting from other causes such as viral hepatitis, alcohol consumption, or other unexplainable reasons [137–139]. In patients with *a priori* elevated transaminases, this is further complicated due to the lack of guidelines as to what contributes a significant increase [138]. In older people, reduced liver size may mean transaminases do not increase as substantially as for younger people [29, 140].

Serum paracetamol concentrations are used to guide treatment in overdose [142]. However, there is still limited evidence on the relationship between therapeutic serum paracetamol concentrations and risk of hepatotoxicity, as in older adults high serum concentrations are not necessarily associated with increased ALT levels [131]. The third stage of clinical paracetamol overdose develops in the next 24 hours, with the symptoms and outcome varying from full recovery to death depending on the severity of the liver damage [101]. Liver biopsy reveals a centrilobularnecrosis, with periportal sparing and little or no inflammatory reaction [93, 134]. In severe cases, acute renal failure may occur [93]. Pharmaco-metabolonomics may help predict individuals at risk of paracetamol hepatotoxicity in the future [143]. 

### 5.7. Management and Treatment of Paracetamol-Induced Liver Injury

Early intervention is essential, as the aim of treatment is to prevent progression to acute liver failure. Paracetamol remains the only hepatotoxin to have effective pharmacotherapy, N-acetylcysteine (NAC), based on well-established nomograms [142]. The benefit of NAC extends those who have developed fulminant hepatic failure [144]. In older people, increased age is associated with increased time to presentation which may be explained in part by the higher proportion of accidental overdose in older patients [128]. By the time older adults present, NAC may no longer be beneficial despite being indicated for late presenters (10–24 hours after overdose) [144]. 

Adjunctive therapy such as corticosteroids or ursodeoxycholic acid is based on anecdotal evidence. The pharmacotherapy of end-stage liver disease (diuretics, beta-blockers) is the same as for other causes of liver disease [144]; however, this is not well described in ageing [86]. Older people do however suffer more ADRs to beta blockers and diuretics [145]. Studies in mice have indicated the usefulness of cimetidine both alone (two doses at 2 and 6hours post paracetamol) and in combination therapy with NAC to reduce ALT/AST concentrations and increase hepatic GSH following paracetamol overdose [146]. Cimetidine may have limited use in hospitalised overdose patients with no effect on ALT/AST being observed if the cimetidine was given after 8 hours after overdose [147]. Additionally, cimetidine has anticholinergic side effects in older adults, which are well known to be associated with poorer functional outcomes [148, 149], further limiting the use of this adjuvant in older patients in clinical care.

## 6. Future Directions

A number of antioxidants have shown promise in protecting against paracetamol-induced liver injury. These include silymarin [150], resveratrol [151], Ukrain [152], *Garcinia kola* seed extract [153], *Ginkgo biloba *extract [154], L-carnitine [155] and oleanic acid [156]. All propose to protect from hepatotoxicity through reduction of oxidative stress mechanisms. However, it must be noted that while these compounds have shown promise in the laboratory setting in animal models, they all suffer the limitation of being given prior to paracetamol overdose. Interestingly, prostagland E2 given either before or 2 hours after paracetamol overdose showed significant hepatoprotective effects in mice [157]. However, their efficacy as a therapy postparacetamol treatment and in humans in the clinical setting still needs to be substantiated.

## 7. Conclusion

Optimal pharmacotherapy is determined when the pharmacokinetics and pharmacodynamics of the drug are understood. However, the age-related changes in pharmacokinetics and pharmacodynamics as well as the increased interindividual variation mean optimal dose selection is a challenge for prescribing in older adults. Paracetamol remains the first-line analgesic of choice for nonmalignant pain; however, dose reduction is mandated for frail older adults despite the pharmacokinetic and pharmacodynamicevidence for such a dose reduction being lacking. Animal studies have indicated a reduction in toxicity in old age, and this may possibly be the same for older frail adults. Understanding how ageing and frailty affect changes in drug clearance and toxicity will improve utilisation of this valuable analgesic and many other medicines by older adults.

## Figures and Tables

**Table 1:** Physiological changes associated with ageing and frailty that can impact on the pharmacokinetics and pharmacodynamics of drugs.

Physiological change	Pharmacokinetic consequences
↑ Gastric pH	*Delay* in absorption no change in the overall
↓ Secretory capacity	*extent*
↓ Gastrointestinal blood flow	
↓ Absorption surface	
↓ Gastrointestinal motility	
↑ Body fat	↑ Vd and t_1/2 _
↓ Lean body mass	↑ Plasma concentration and ↓ Vd of hydrophilic
	drugs
↓ Total body water	↑ Free fraction of highly protein-bound acidic
↓ Serum albumin	drugs
↑*α*1-acid glycoprotein	↓ Free fraction of basic drugs

↓ Hepatic blood flow	↓ First-pass metabolism
↓ Hepatic mass	Phase I metabolism of some drugs may be slightly
↓ CYP content	impaired
	↓/↔ Phase II in fit older adults, ↓in frail
	?/↓ Phase III
Pseudocapillarisation of the liver	Impaired transfer of chylomicrons and possibly
sinusoidal endothelium	medications from sinusoid to space of Disse

↓ Renal blood flow and glomerular	Renal elimination of drugs can be impaired
filtration rate	altering drug half-life
↓ Tubular secretion	

Physiological change	Pharmacodynamic consequences

↓ Blood supply to brain	↑ Sensitivity to centrally acting drugs such as
↓ Baroreceptor activity	benzodiazepines

↓ Resting heart rate, stroke volume, and	↓ Response to beta blockers such as metoprolol
cardiac output	

↓ Plasma renin ↓ Urine aldosterone	

↓ Hepatic GSH	↓ Detoxification ability of the liver
Dysregulation of Kupffer cells	Dysregulation of immune response to drugs and
Dysregulation of the immune system	other toxins
Mitochondrial dysregulation	↑ Susceptibility to DILI

↓, decreased; ↑, increased; ↔, no change; ?, unknown; CYP, Cytochrome P450; Vd, volume of distribution; t_1/2_, half-life; DILI, drug-induced liver injury; GSH, glutathione; adapted from [13] and references [2, 11–23].

**Table 2:** Changes in the cytochrome P450 activity with ageing.

CYP Enzyme	Change with ageing	Probe drug used	Confounding factors
CYP1	↓	Theophylline	Ethnic polymorphisms, sex differences, lifestyle, and disease

CYP2CYP2C9CYP2C19	↓ (~25%)	PhenytoinWarfarinOmeprazole	Age-related effects, and unrecognised environmental effects, and pharmacogenetic variation

CYP2D6	↓ Older women↓ Older Japanese men	DextromethorphanHaloperidol	Genetic polymorphisms

CYP2E1	↓ Aged rats*↔* Aged mice↓ Activity in human liver microsomes*↔* Human hepatocytes	Chlorzoxazone	? Gender-conflicting results Polymorphisms

CYP3ACYP3A4	↓ Aged rodents*↔* Humans↑ CL in women, no age effects	Cyclosporine, Erythromycin, Verapamil, Midazolam	InducersInducers
Multiple	↓	Antipyrine	Metabolised by CYP3A4, 1A2 and 2C8/9

CYP, cytochrome P450; ↓, decreased; ↑, increased; *↔*, no change; ?, unknown; CL, clearance; adapted from [13, 52–55].

**Table 3:** Selected reports of paracetamol-related hepatotoxicity, deaths, and transplantsin the United States, Canada, United Kingdom, Malaysia, and Australia for the period 1989–2010. Only studies that have included a sub grouping for “older adults”, defined as those aged > 60 years, are included.

Source	Approximate population Size	Cases/million population/year	% of Reports for those aged>60 years	% of Reports unintentional	Reference
Spontaneous ADR reports, AUS1990–2010	17–22.5 million	0.04 deaths	37.5% deaths	NR	Pers. Comm. Graeme Harris, ACSOM, 24/8/2010

Ballarat Hospital Records, AUS 2000–2003	0.2 million	240 hospitalisations	2.6% hospitalisations	4.7%	[56]

Penang General Hospital, Malaysia 2000–2002	Approx 1.3 million	42.3 cases of poisoning	1.2% of poisoning cases	33.3%	[57]

Calgary, Canada1995–2004	1.1 million	140.2 hospitalisations	4.5% hospitalisations	13%	[58]

US Transplant Centres1998–2001	17 tertiary care centres	NR	6% ALFs6.8% deaths	57% ALFs	[59]

US 1990–2001	250 million	1.83 deaths	4% hospitalisations 14% deaths	23 % hospitalisations22% deaths	[60]

Cardiff, UK 1989–2002	Approx 2.9 million	185 hospital admissions	1.6 % of admissions in adults 60–69 years1.8% of admissions in adults >70 years	All intentional	[61]

England and Wales 1993–1998	NR	15720 deaths, 13% due to paracetamol alone, 5.8% due to paracetamol and other drugs	11.5% deaths per million males during 1993–199814.2% deaths per million females during 1993–1998	NR	[62]

NR, not reported; US, United States; UK, United Kingdom; AUS, Australia; ADR, adverse drug reaction; ACSOM, Advisory Committee on the Safety of Medicines; ALF, acute liver failure; APAP, paracetamol.
